# Lipid alternations in the plasma of COVID-19 patients with various clinical presentations

**DOI:** 10.3389/fimmu.2023.1221493

**Published:** 2023-08-29

**Authors:** Xiao Liang, Xin Qi, Jin Yang, Xiaorui Wang, Hongyu Qin, Fang Hu, Han Bai, Yixin Li, Chengsheng Zhang, Bingyin Shi

**Affiliations:** ^1^ Cancer Center, The First Affiliated Hospital of Xi’an Jiaotong University, Xi’an, China; ^2^ Precision Medicine Center, The First Affiliated Hospital of Xi’an Jiaotong University, Xi’an, China; ^3^ Department of Medical Oncology, The First Affiliated Hospital of Xi’an Jiaotong University, Xi’an, China; ^4^ The MED-X Institute, The First Affiliated Hospital of Xi’an Jiaotong University, Xi’an, China; ^5^ The Jackson Laboratory for Genomic Medicine, Farmington, CT, United States; ^6^ Department of Endocrinology, The First Affiliated Hospital of Xi’an Jiaotong University, Xi’an, China

**Keywords:** SARS-CoV-2, COVID-19, asymptomatic infection, lipids, long-term nucleic acid test positive

## Abstract

**Background:**

COVID-19 is a highly infectious respiratory disease that can manifest in various clinical presentations. Although many studies have reported the lipidomic signature of COVID-19, the molecular changes in asymptomatic severe acute respiratory syndrome coronavirus 2 (SARS-CoV-2)-infected individuals remain elusive.

**Methods:**

This study combined a comprehensive lipidomic analysis of 220 plasma samples from 166 subjects: 62 healthy controls, 16 asymptomatic infections, and 88 COVID-19 patients. We quantified 732 lipids separately in this cohort. We performed a difference analysis, validated with machine learning models, and also performed GO and KEGG pathway enrichment analysis using differential lipids from different control groups.

**Results:**

We found 175 differentially expressed lipids associated with SASR-CoV-2 infection, disease severity, and viral persistence in patients with COVID-19. PC (O-20:1/20:1), PC (O-20:1/20:0), and PC (O-18:0/18:1) better distinguished asymptomatic infected individuals from normal individuals. Furthermore, some patients tested positive for SARS-CoV-2 nucleic acid by RT-PCR but did not become negative for a longer period of time (≥60 days, designated here as long-term nucleic acid test positive, LTNP), whereas other patients became negative for viral nucleic acid in a shorter period of time (≤45 days, designated as short-term nucleic acid test positive, STNP). We have found that TG (14:1/14:1/18:2) and FFA (4:0) were differentially expressed in LTNP and STNP.

**Conclusion:**

In summary, the integration of lipid information can help us discover novel biomarkers to identify asymptomatic individuals and further deepen our understanding of the molecular pathogenesis of COVID-19.

## Introduction

At the end of 2019, an acute respiratory disease caused by SARS-CoV-2 continued to spread rapidly all over the world and attracted extensive attention (1). Coronavirus disease 2019 (COVID-19) is a highly contagious disease that targets the respiratory tract. Asymptomatic infections (AS) are those who do not have any symptoms but carry SARS-CoV-2 (2). The number of asymptomatic subjects has raised concerns worldwide because they are difficult to identify. In addition, some of the patients tested positive for the nucleic acid of SARS-CoV-2 by RT-PCR but did not become negative for a longer period of time (≥60 days, herein designated as long-term nucleic acid test positive, LTNP) whereas others tested negative for the viral nucleic acid in a shorter period of time (≤45 days, designated as short-term nucleic acid test positive, STNP). Asymptomatic and LTNP COVID-19 patients both threaten global public health. However, little is known about the detailed mechanisms responsible for the LTNP and STNP as well as AS and health controls.

At present, research on virus–host interaction generally focuses on proteomic changes. However, metabolites, as the final product of cellular processes, provide different insights into the mechanism of viral infection (3).. Previous studies have shown that the pathogenesis of viral infection is closely related to the lipid metabolism of infected cells. 25-Hydroxycholesterol (25-HC) inhibits viral invasion by inducing inflammatory and immune responses (4). It has been found that the replication of SARS-CoV and SARS-CoV-2 is inhibited by 25-HC (5). Omega-3 PUFA-derived lipid mediator protectins have been shown to inhibit influenza virus replication by blocking viral mRNA output (3).

Lipids play an important role in the life cycle of viruses. Their involvement in virus infection includes fusion of virus membranes and host cells, virus replication, endocytosis, and exocytosis (6). SARS-CoV-2 is an enveloped virus surrounded by a lipid bilayer. Viruses manipulate host cells by targeting lipid synthesis and signal transduction pathways. They modify the host cells, enveloping them to produce lipids (7). Therefore, understanding the alterations in host cell lipids can be beneficial in identifying potential biomarkers that can distinguish between healthy individuals, those with asymptomatic infections, and individuals with different types of symptoms caused by SARS-CoV-2. This knowledge can further aid in the development of targeted treatments.

In this study, we aimed to investigate the potential pathogenesis of COVID-19 by analyzing the host response to SARS-CoV-2 infection in humans. We conducted lipidomics analysis on plasma samples obtained from a total of 220 individuals, including 104 COVID-19 patients and 62 healthy controls. We identified 175 differentially expressed lipids that were associated with the SASR-CoV-2 infections, disease severity, and viral persistence in the COVID-19 patients. For example, we found that 81 lipids showed significant differences between AS and healthy controls, and 15 lipids could distinguish between LTNP and STNP. Moreover, we performed machine learning to further identify and verify the potential biomarkers for different disease severity. These striking results provided the potential biomarkers to learn about the mechanism of COVID-19, particularly in relation to AS, LTNP, and STNP. Furthermore, these findings have the potential to identify diagnostic and treatment targets for SARS-CoV-2 infection.

## Methods

### Ethics statement

This study was approved by the Ethics Committee of the First Affiliated Hospital of Xi’an Jiaotong University (XJTU1AF2020LSK-015) and the Renmin Hospital of Wuhan University (WDRY2020-K130). All participants enrolled in this study provided written informed consent by themselves or their surrogates. The high throughput sequencing of plasma samples was performed on existing samples collected during standard diagnostic tests, posing no extra burden to patients.

### Case definition and study cohort

The definition and classification of all COVID-19 patients in this study follow the Guidelines of the World Health Organization and the “Guidelines on the Diagnosis and Treatment of the Novel Coronavirus Infected Pneumonia” developed by the National Health Commission of People’s Republic of China (8–10). This study cohort included 220 plasma samples derived from 166 individuals, consisting of healthy controls (HC, n=62), asymptomatic infections (AS, n=16), and symptomatic patients (SM, n=88). Symptomatic patients consisted of moderate diseases (MD, n=42) and severe diseases (SD, n=46). Then according to the time of a positive nucleic acid test, the individuals in the SM group were divided into 17 long-term nucleic acid test positive (LTNP, ≥60 days) and 34 short-term nucleic acid test positive (STNP, ≤45 days) individuals. In this study, based on the clinical observation that most of the COVID-19 patients hospitalized in the Renmin Hospital in Wuhan tested negative for the nucleic acid test within 45 days, we therefore defined the STNP as ≤45 days whereas the LTNP was ≥60 days. The demographic features, clinical laboratory testing results and other relevant information are provided in **Supplementary Table 1**.

### Blood sample collection and plasma preparation

The peripheral blood was collected into the standard EDTA-K2 Vacuette Blood Collection Tubes (Jiangsu Yuli Medical Equipment Co., Ltd, China; Cat.Y09012282) and stored at room temperature or 4°C until processed, and the sample storage time did not exceed 12 hours. The plasma was prepared after centrifugation of the whole blood sample at 2500 rpm for 20 minutes and stored in the -80°C freezer until used for the studies. All the experimental procedures were completed inside a biosafety level 2 (BSL-2) laboratory at the Department of Clinical Diagnostic Laboratories, Renmin Hospital of Wuhan University. A total of 220 plasma samples were collected from 62 HC and 104 COVID-19 at different time-points from some of these individuals.

For lipid compounds, samples were thawed on ice, whirled for around 10s, and then centrifuged for 3000 rpm at 4°C for 5 min. We took 50 μL of one sample and homogenized it with 1 mL mixture (include methanol, MTBE and internal standard mixture). We whirled the mixture for 2 min and then added 500μl of water and whirled the mixture for 1 min and centrifuged it for 12,000 rpm at 4°C for 10 min. We extracted 500 μL of supernatant and concentrated it. We dissolved powder with 100 μL mobile phase B and then stored it at -80°C. Finally, we moved the dissolving solution into the sample bottle for LC-MS/MS analysis.

### HPLC conditions

For lipid compounds, the sample extracts were analyzed using an LC-ESI-MS/MS system (UPLC, Shim-pack UFLC SHIMADZU CBM 30A system, https://www.shimadzu.com/; MS, QTRAP^®^ System, https://sciex.com/). The analytical conditions were as follows UPLC: column, Waters ACQeITY UPLC HSS T3 C18 (1.8 µm, 2.1 mm*100 mm); column temperature, 40°C; flow rate, 0.4 mL/min; injection volume, 5μL; solvent system, water (0.04% acetic acid): acetonitrile (0.04% acetic acid); gradient program, 95:5 V/V at 0 min, 5:95 V/V at 11.0 min, 5:95 V/V at 12.0 min, 95:5 V/V at 12.1 min, 95:5 V/V at 14.0 min. The effluent was alternatively connected to an ESI-triple quadrupole-linear ion trap (QTRAP)-MS.

### ESI-Q TRAP-MS/MS

LIT and triple quadrupole (QQQ) scans were acquired on a triple quadrupole-linear ion trap mass spectrometer (QTRAP), QTRAP^®^ LC-MS/MS System, equipped with an ESI Turbo Ion-Spray interface, operating in positive and negative ion mode, and controlled by Analyst 1.6.3 software (Sciex). For lipid compounds, the following applied: source temperature 550 °C; ion spray voltage (IS) 5500 V (positive), -4500 V (negative); ion source gas 1 (GS1), gas 2 (GS2), curtain gas (CUR) were set at 55, 60, and 25 psi, respectively. The collision gas (CAD) was high. Instrument tuning and mass calibration were performed with 10 and 100 μmol/L polypropylene glycol solutions in QQQ and LIT modes, respectively. DP and CE for individual MRM transitions was done with further DP and CE optimization. A specific set of MRM transitions were monitored for each period according to the metabolites eluted within this period in both hydrophilic and hydrophobic compounds.

### Statistical analysis and machine learning

The mass spectrum data were processed in Analyst 1.6.3 software. The characteristic ions of each substance were screened by QQQ. The MultiQuant software was used to open the mass spectrum file of the samples, and the chromatographic peaks detected by each metabolite in different samples were integrated and corrected according to the retention time and peak type information of metabolites. The statistical analysis was performed using the first blood sample collected from the participants. Orthogonal Partial Least Squares-Discriminant Analysis (OPLS-DA) was used to evaluate the statistical significance in different groups. Metabolites were selected using the following criteria: a variable importance in projection (VIP) value ≥ 1 and Fold change (FC)≥1.5 or ≤0.67. The normalized quantitation of metabolites was used as modeling data for every two compared groups. Then, 75% of the modeling data was selected as the training cohort, and the rest was used in the testing cohort. From the training set, we selected important lipids *via* machine learning using the xgboost method with the R package “xgboost”(version 1.4.1.1) (11). For analysis of the five classes, 28 subclasses, carbon chain length, and degree of saturation of lipids in five compared groups, we used independent t-tests when the data were normally distributed and homoscedasticity; otherwise, the Mann-Whitney was used. The statistical analyses were performed in SPSS version 18.0 software. The data statistics used the mean values of raw intensities of lipid molecules with five classes, 28 subclasses, and different lengths of carbon chain (unsaturation) in different classes.

## Results

### Lipid profiling of COVID-19 plasma

In this study, lipidomics analysis was conducted on 220 plasma samples obtained from a total of 166 individuals. The sample distribution included 62 healthy controls, 16 asymptomatic infections, 42 individuals with moderate diseases, and 46 individuals with severe diseases. Additionally, there were repeated samples from 5 healthy controls, 12 individuals with moderate diseases, and 23 individuals with severe diseases, which were collected twice or more. The detailed descriptions of 166 individuals are shown in **Table S1**. We used OPLS-DA to analyze the 220 plasma samples. In total, we identified five major lipid classes [fatty acyls (FA), glycerophospholipids (GP), glycerolipids (GL), sphingolipids (SP), and sterol lipids (ST)] containing 28 lipid subclasses [carbocyclic fatty acids (CAR), free fatty acid (FFA), diglyceride (DG); monoglyceride (MG), lyso-phosphatidic acid (LPA), lyso-phosphatidylcholine (LPC), triglyceride (TG), ether-linked lyso-phosphatidyl-cholines (LPC-O), lyso-phosphatidylethanolamine (LPE), lyso-phosphatidylglycerol (LPG), lyso-phosphatidylinositol (LPI), lyso-phosphatidylserine (LPS), phosphatidic acid (PA), phosphatidylcholine (PC), cholesterylesters (CE), ether-linked phosphatidyl-cholines (PC-O), phosphatidylethanolamine (PE), phosphatidylglycerol (PG), phosphatidylinositol (PI), phosphatidylserine (PS), ceramides (Cer/Cert/Cerm), ceramides phosphate (CerP), and sphingomyelin], totaling 732 lipid molecules; detailed information of lipids is listed in **Table S2**. We analyzed differentially expressed lipids (DELs) in five compared groups, including COV vs HC, AS vs HC, SM vs AS, SD vs MD, and LTNP vs STNP (**Table S3**).

### Lipids associated with SARS-COV-2 infection

**Figure 1:**
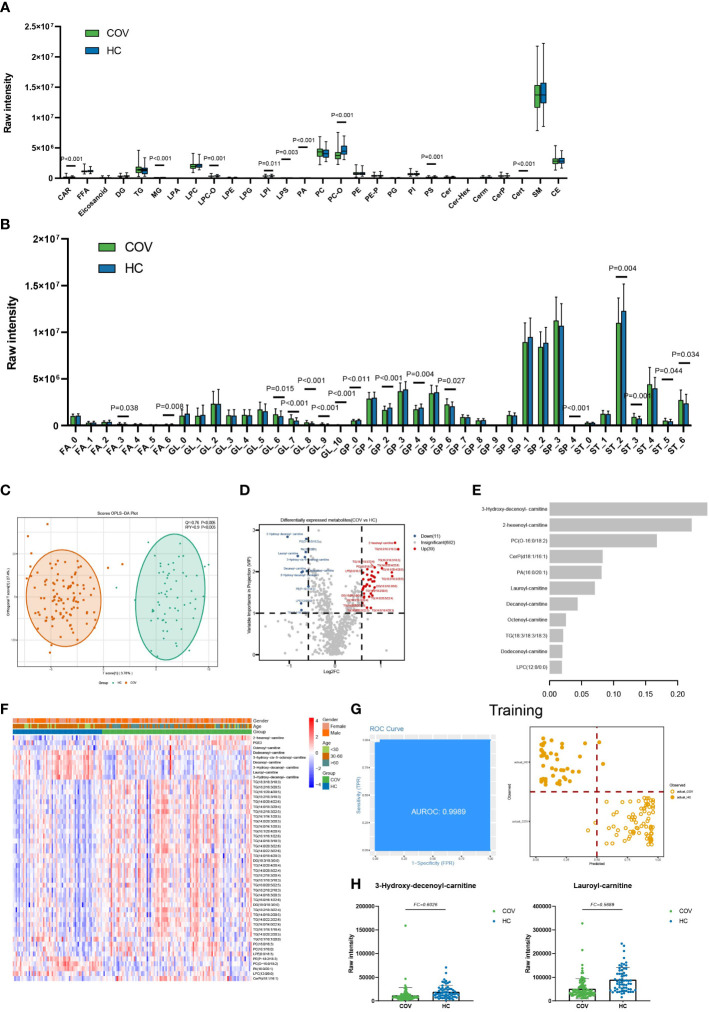
Lipids associated with SARS-COV-2 infections. **(A)** The raw intensity of 28 subclasses of lipids in COV vs HC. **(B)** The raw intensity of different degrees of unsaturation for FA, GL, GP, SP, and ST classes in COV vs HC. **(C)** The OPLS-DA scores plot of COV vs HC. **(D)** the volcano plot of COV vs HC. Red dots represent the upregulated lipids (FC≥1.5, VIP≥1); blue dots represent the downregulated lipids (FC ≤ 0.67, VIP≥1); gray dots represent lipids without significant changes (0.67<FC<1.5, VIP<1). **(E)** The important lipids prioritized by xgboost analysis. **(F)** The heatmap of 50 differentially expressed lipids in COV vs HC. **(G)** Receiver operating characteristic (ROC) and performance of the xgboost model in the training set. **(H)** the raw intensity of 3-Hydroxy-decenoyl-carnitine and Lauroyl-carnitine.

The 50 lipids were at significantly different levels in COVID-19 patients compared to healthy controls (VIP ≥ 1 and FC ≥ 1.5 or FC ≤ 0.67) (**Figures 1C, D**). Further, the DELs were used to perform the KEGG pathway analysis. The result suggested that the KEGG pathways of DELs were mainly involved in glycerophospholipid metabolism (hsa00564) and glycerolipid metabolism (hsa00561) (**Supplementary Figure 5A**).

Moreover, compared with the healthy controls, 9 of 28 lipids subclasses showed a statistical difference in COVID-19 patients, including LPS, LPI, LPC-O, PA, PS, PC-O, CAR, Cert, and MG (**Figure 1A**). In addition, GL with unsaturation greater than 5, GP with unsaturation of 6, and ST with unsaturation of 3, 5, and 6 showed high average levels in COV (**Figure 1B**). GP with unsaturation of 0, 2, and 4, as well as ST with unsaturation of 2, showed low average levels in COV. Our statistical results indicate that short-chain FA and GP, FA (C=14), GL (C=32), GP (C=34), and ST (C=18) show low average levels in COV. FA (C=12) and long-chain ST show high average levels in COV (**Supplementary Figure 3A**).

We built an xgboost machine learning model based on intensity of DELs from 62 HC and 104 COVID-19 patients, leading to prioritization of 11 important lipids (**Figure 1E**). This model reached an area under the curve (AUC) of 0.9989 in the training set, and only 4 of a total of 124 samples (accuracy=0.9677) were classified incorrectly (**Figure 1G**). In the test set, 37 of 42 were correctly classified (accuracy=0.8810), and the AUC of this model can reach 0.9435 (**Supplementary Figure 4**).

### Lipids associated with asymptomatic infections of SARS-CoV-2

**Figure 2:**
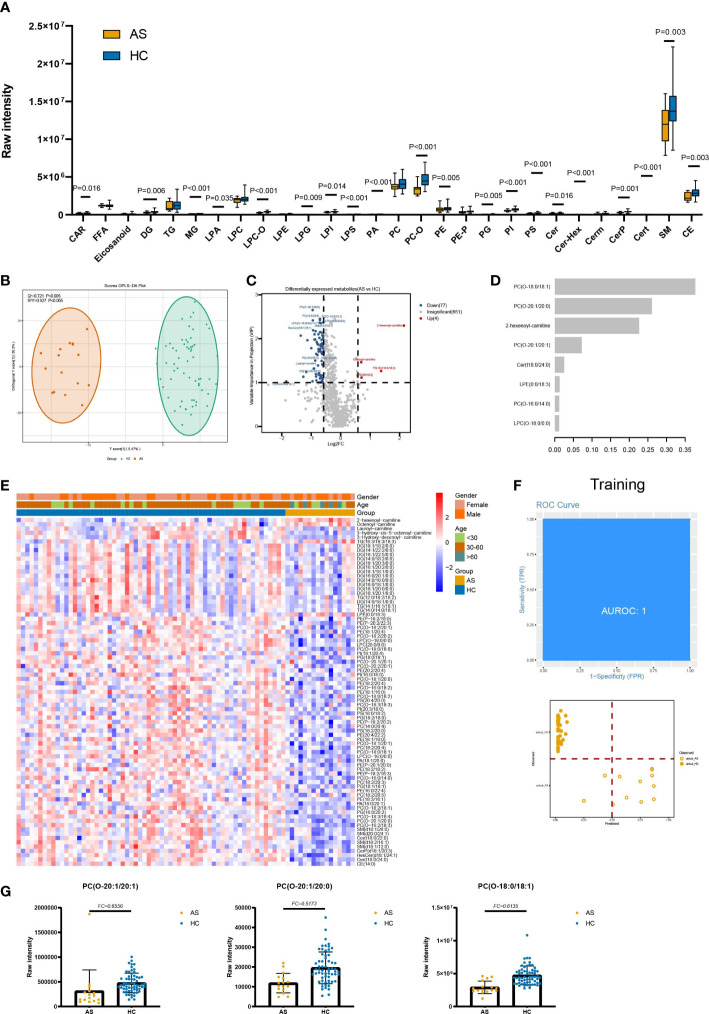
Lipids associated with asymptomatic infection of SARS-COV-2. **(A)** The raw intensity of 28 subclasses of lipids in AS vs HC. **(B)** The OPLS-DA scores plot of AS vs HC. **(C)** the volcano plot of AS vs HC. Red dots represent the upregulated lipids (FC≥1.5, VIP≥1); blue dots represent the downregulated lipids (FC ≤ 0.67, VIP≥1); gray dots represent lipids without significant changes (0.67<FC<1.5, VIP<1). **(D)** The important lipids prioritized by xgboost analysis. **(E)** The heatmap of 81 differentially expressed lipids in AS vs HC. **(F)** Receiver operating characteristic (ROC) and performance of the xgboost model in the training set. **(G)** the raw intensity of PC(O-20:1/20:1), PC(O-20:1/20:0) and PC(O-18:0/18:1).

For AS vs HC, we selected a total of 81 DELs (VIP ≥ 1 and FC ≥ 1.5 or FC ≤ 0.67) (**Figures 2B, C**). The KEGG pathways analysis using DELs suggested that pathways were mainly involved in glycerophospholipid metabolism (hsa00564) and glycerolipid metabolism (hsa00561) (**Supplementary Figure 5B**).

Notably, in individuals with AS, there were significant decreases observed in various subclasses of lipids. Specifically, the SP subclasses, including Cer, Cert, CerP, Cer-Hex, and sphingomyelin; the ST subclasses, including CE; GP subclasses, including PS, PA, PI, PE, PG, PC-O, LPI, LPA, LPS, LPG, and LPC-O; the GL subclasses, including DG and MG; and the FA subclass, including CAR, showed marked decreases when compared to HC (**Figure 2A**). Compared to HC, the average levels of almost all GP with different unsaturation degrees in AS are significantly downregulated. At the same time, the average levels of ST and SP with unsaturation degrees equal to 1 and 2 are lower in AS (**Supplementary Figure 2A**). In terms of chain length, the average levels of medium-chain and long-chain GP, medium-chain GL, short-chain ST, ST (C=18), and FA (C=14) are significantly lower in the AS group (**Supplementary Figure 3B**). As for SP, the average levels of short-chain, medium-chain, and long-chain are all low in AS.

We built an xgboost machine learning model based on intensity of DELs from 62 HC and 16 asymptomatic infections, leading to prioritization of eight important lipids (**Figure 2D**). This model reached an AUC of 1 in the training set, and two of the training set samples (accuracy=0.9655) were classified incorrectly (**Figure 2F**). In the test set, 18 of 20 were correctly classified (accuracy=0.9000) (**Supplementary Figure 4**) and the AUC is 0.9297.

### Lipids associated with symptomatic infection of SARS-CoV-2

**Figure 3:**
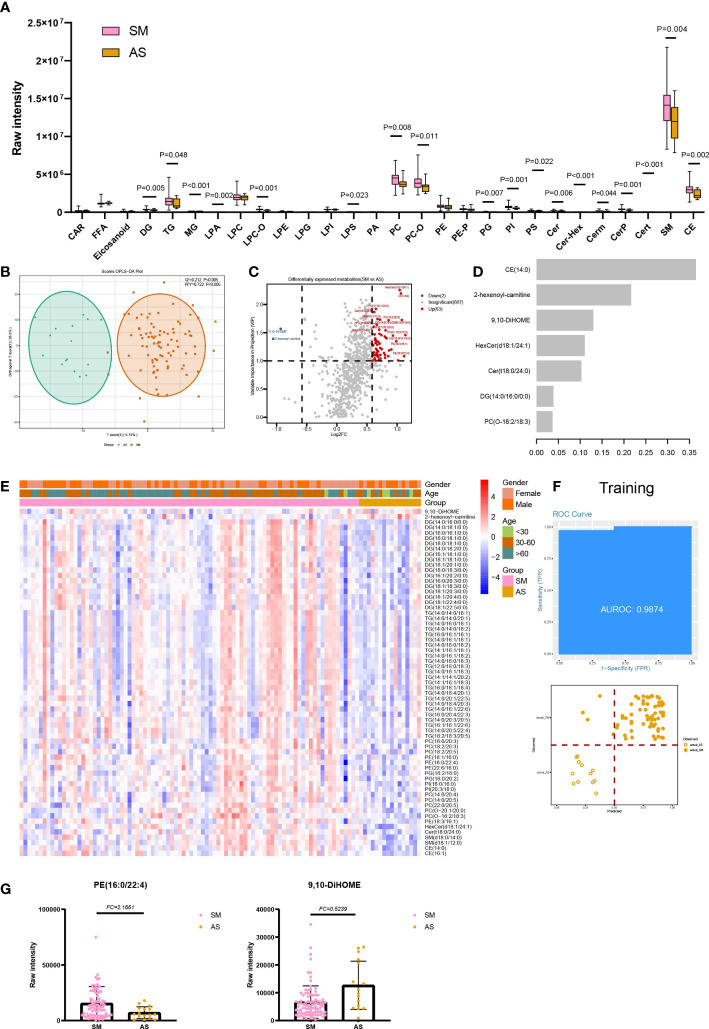
Lipids associated with asymptomatic and symptomatic COVID-19. **(A)** The raw intensity of 28 subclasses of lipids in SM vs AS; **(B)** The OPLS-DA scores plot of SM vs AS. **(C)** the volcano plot of SM vs AS. Red dots represent the upregulated lipids (FC≥1.5, VIP≥1); blue dots represent the downregulated lipids (FC ≤ 0.67, VIP≥1); gray dots represent lipids without significant changes (0.67<FC<1.5, VIP<1). **(D)** The important lipids prioritized by xgboost analysis. **(E)** The heatmap of 65 differentially expressed lipids in SM vs AS. **(F)** Receiver operating characteristic (ROC) and performance of the xgboost model in the training set. **(G)** the raw intensity of PE(16:0/22:4) and 9,10-DiHOME.

The lipids were divided into five classes, four (GL, GP, SP, and ST) of which show statistical significance in SM and AS (**Supplementary Figure 1A**). Then, the 28 subclasses of five lipids classes were analyzed to explore the lipid subclasses differences and consisted of the results of five classes, CE, and all subclasses of SP (Cer, Cer-Hex, Cerm, CerP, Cert, and sphingomyelin), and GL (DG, TG, and MG) exhibiting differences in SM and AS (**Figure 3A**). Besides, 8 of 15 SP subclasses showed a significant difference in SM and AS, including LPA, LPC-O, LPS, PC, PC-O, PG, PI, and PS. We also found that lipids with different carbon chain lengths and degrees of unsaturation show significant changes in SM and AS. Compared to AS, there are statistical differences in the unsaturation of GP, GL, SP, and ST in both SM and AS (**Supplementary Figure 2B**). The average levels of short-, medium-, and long-chain GL, GP, SP, and ST are significantly upregulated in SM (**Supplementary Figure 3C**).

Through OPLS-DA (VIP ≥1) and fold change (FC ≥ 1.5 or FC ≤ 0.67), we discovered a total of 65 differentially expressed lipids in SM and AS (**Figures 3B, C**, **E**; **Table S3**). To research the function of lipids, six significant KEGG pathways were found, including glycerophospholipid metabolism (hsa00564) and glycosylphosphatidylinositol (GPI)-anchor biosynthesis (hsa00563) (**Supplementary Figure 5**; **Table S4**). In addition, an xgboost machine learning model was built based on the 65 DELs derived from the 88 symptomatic infections and asymptomatic infections, leading to prioritization of seven lipids (**Figure 3D**). This model reached an AUC of 0.9874 and an accuracy of 0.8267 in the training set (**Figure 3F**). We then tested the model on the test cohort, and the AUC value was 0.9545 (**Supplementary Figure 4**).

### Lipids associated with disease severity of COVID-19

**Figure 4:**
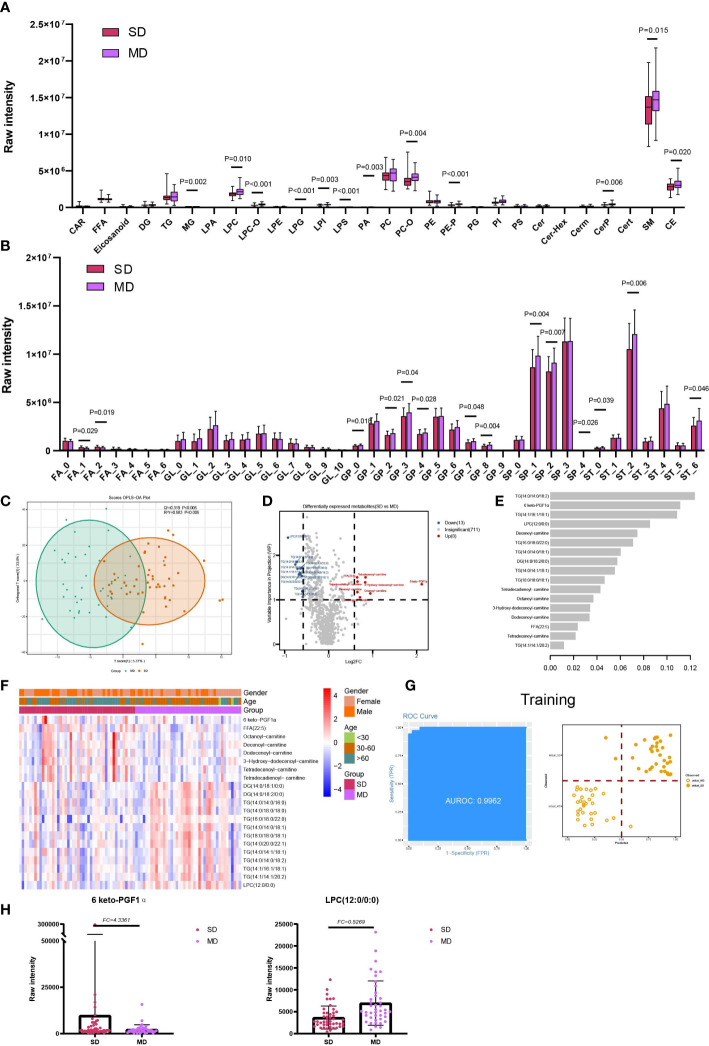
Lipids associated with the disease severity of COVID-19. **(A)** The raw intensity of 28 subclasses of lipids in SD vs MD; **(B)** The raw intensity of different degrees of unsaturation for FA, GL, GP, SP and ST classes in SD vs MD; **(C)** the OPLS-DA scores plot of SD vs MD. **(D)** the volcano plot of SD vs MD. Red dots represent the upregulated lipids (FC≥1.5, VIP≥1); blue dots represent the downregulated lipids (FC ≤ 0.67, VIP≥1); gray dots represent lipids without significant changes (0.67<FC<1.5, VIP<1). **(E)** The important lipids prioritized by xgboost analysis. **(F)** The heatmap of 21 differentially expressed lipids in SD vs MD. **(G)** Receiver operating characteristic (ROC) and performance of the xgboost model in the training set. **(H)** the raw intensity of 6 keto-PGF1α and LPC(12:0/0:0).

Significant differences were observed in three out of five lipid classes between SD and MD groups. These were GP (P=0.032), SP (P=0.014), and ST (P=0.020) (**Supplementary Figure 1A**). Furthermore, among the 28 lipid subclasses, 12 showed statistically significant differences between the SD and MD groups. These subclasses were MG, LPC, LPC-O, LPG, LPI, LPS, PA, PC-O, PE-P, CerP, sphingomyelin, and CE. (**Figure 4A**). The statistical results of unsaturation indicate that most FA and ST have low unsaturation levels (unsaturation ≤ 3). Additionally, FAs with unsaturation levels of 1 and 2 have higher average levels in the SD group, while monounsaturated and polyunsaturated GPs and SPs, as well as STs with unsaturation levels of 0 or 6, have higher average levels in the MD group (**Figure 4B**).Furthermore, our statistical analysis shows that compared to the SD group, the MD group has higher average levels of long-chain and short-chain GP and SP, SP (C=32), and ST (C=18) (**Supplementary Figure 3D**). On the other hand, the SD group has a higher average level of long-chain FA (**Supplementary Figure 3D**).

For the detailed compounds, we found 21 DELs in SD and MD *via* OPLS-DA (VIP ≥1) and FC (FC ≥ 1.5 or FC ≤ 0.67) (**Figures 4C, D**; **Table S3**). Furthermore, these DELs were mainly enriched in glycerolipid metabolism (hsa00561) and glycerophospholipid metabolism (hsa00564) (**Supplementary Figure 5**; **Table S4**). Based on the significant lipids, the xgboost model was established using 17 lipids with high importance scores (**Figure 4E**). This model reached an AUC of 0.9962, and the accuracy of the training set samples was 0.9076 (**Figure 4G**). We tested the model on the test cohort, and the AUC value was 0.8939 (**Supplementary Figure 4**).

### Lipids associated with the viral persistence of SARS-CoV-2 infection

**Figure 5:**
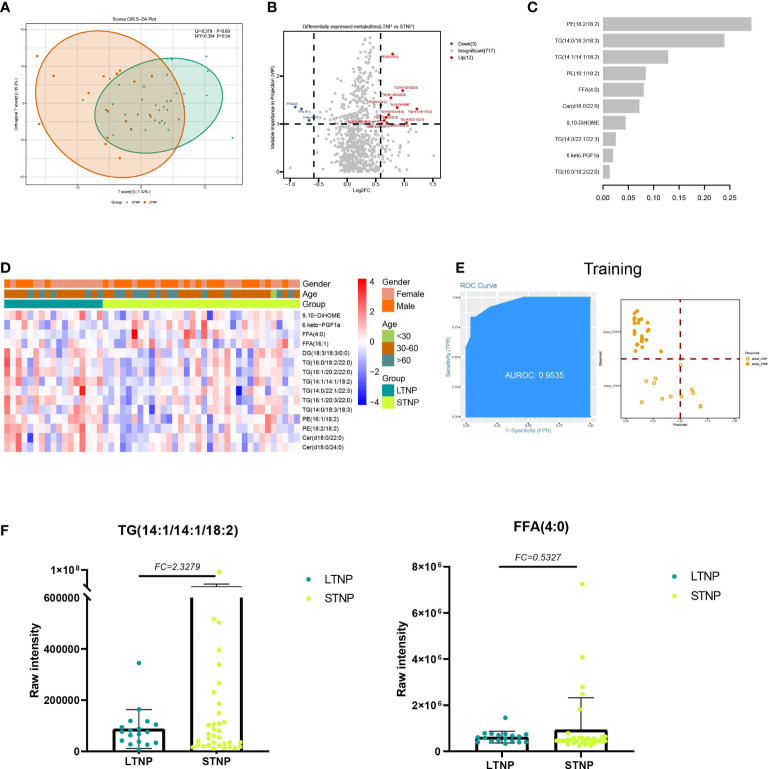
Lipids associated with the duration of a positive nucleic acid test for SARS-CoV-2 infection. **(A)** The OPLS-DA scores plot of LTNP vs STNP. **(B)** the volcano plot of LTNP vs STNP. Red dots represent the upregulated lipids (FC≥1.5, VIP≥1); blue dots represent the downregulated lipids (FC ≤ 0.67, VIP≥1); gray dots represent lipids without significant changes (0.67<FC<1.5, VIP<1). **(C)** The important lipids prioritized by xgboost analysis. **(D)** The heatmap of 15 differentially expressed lipids in LTNP vs STNP. **(E)** Receiver operating characteristic (ROC) and performance of the xgboost model in the training set. **(F)** the raw intensity of TG (14:1/14:1/18:2) and FFA (4:0).

For LTNP vs STNP, we identified four classes (FA, GL, GP and SP) and six subclasses (Eicosanoid, FFA, PE, Cer, DG, and TG) totaling 15 differentially expressed lipids, including 12 upregulated and 3 downregulated lipids (VIP ≥ 1 and FC ≥ 1.5 or FC ≤ 0.67) (**Figure 5B**). In addition, we found that in the STNP group, there were higher average levels of ST with a carbon chain length of 20 as well as ST with an unsaturation value of 4 (**Supplementary Figure 3D**). Furthermore, KEGG results showed that these DELs were enriched in the glycerolipid metabolism (hsa00561) and glycerophospholipid metabolism pathways (hsa00564) (**Supplementary Figure 5**; **Table S4**).

We also supposed DELs could be used as predictor for LTNP patients from STNP patients. A total of 17 LTNP and 34 STNP patients were included in this analysis. We built an xgboost machine learning model based on 15 DELs and select 10 Lipids with high importance scores (**Figures 5A, C, D**). This model reached an AUC of 0.9535, and the accuracy was 0.8421 in the training set (**Figure 5E**). We then tested the model on an independent cohort of five LTNP and nine STNP patients; the AUC value was 0.7667 (**Supplementary Figure 5**).

## Discussion

In this study, we applied LC-ESI-MS/MS to analyze the lipid profiles of 220 plasma samples. They were also divided into five control groups and found that lipidome is highly dynamic in different groups. In total, 732 lipids were found in our analysis (**Table S2**), and 175 DELs were detected in five compared groups (**Table S3**). In addition, combined with machine learning, multiple biomarkers were identified to distinguish the different COVID-19 severity from healthy controls.

Compared with HC, though the raw intensity of five lipid classes showed no obvious change, the subclasses represented marked transformation in COV. To further explore GP in COV, we found that GP subclasses like LPS and PS are both downregulated in COV (**Figure 1A**).

It was reported that PS was a global immunosuppressive signal in efferocytosis, infectious disease, and cancer (12). LPS could increase endogenous TNF-α expression, generating tolerogenic dendritic cells to exhaust effector CD4 T cells (13, 14). The possible explanation of the relatively lower intensity level of PS in COV might reflect the immune response in SARS-COV-2 infections. We also explored the unsaturation degree and carbon-chain length influence of FA in COV; we found fewer short-chain FA (SCFAs) in COV (**Supplementary Figure 3A**). Dr. Takabayashi cultured nasal epithelial cells and exposed them to SCFA and saw that the SCFAs had suppressed ACE2 expression (15). As is known, RBD of SARS-COV-2 can bind to ACE2 to enter the host cell. For COVID-19 patients, downregulated SCFAs might encourage binding between ACE2 and SARS-COV-2.

Combined with the fold change and machine learning result, acylcarnitine like 3-Hydroxy-decenoyl-carnitine (FC=0.49, C=12) and Lauroyl-carnitine (FC=0.57, C=12) play an important role in distinguishing COV and HC (**Figure 1H**). We also found the acylcarnitine intensity is obviously different in COV compared to HC (**Figure 1F**). Acylcarnitines serve as carriers to transport activated long-chain fatty acids into mitochondria for β-oxidation (16) as energy powerhouse for cell activities. For COVID-19 patients, downregulated acylcarnitines may suggest that infected people try to prevent virus replication by reducing ATP production, which is consistent with our result that show there are no obvious changes in long-chain FA (**Figure 1B**).

In AS vs HC, raw intensity of GP, SP, and ST presented lower levels in AS. It is also disclosed that 20 of total 28 detected lipid subclasses show marked changes in AS. Though there are no obvious clinical symptoms in AS, the lipid profile changes dramatically (**Figure S1A**). For example, SP metabolites have key roles in the regulation of both trafficking and functions of immune cells (17), and decreased levels of SP and GP were observed in both non-severe and severe COVID-19 patients. In the DELs, the fold change of 2-hexenoyl-carnitine (FC=4.42, C=6) is the highest. Unexpectedly, unlike downregulated acylcarnitines in COV vs HC, 2-hexenoyl-carnitine raw intensity is upregulated in AS, which is also present in COV vs HC DELs. One possible explanation is that the short-chain acylcarnitines are metabolites of lipids, and amino acids and carbohydrates are released in plasma (18).The features, such as PC(O-20:1/20:1), PC(O-20:1/20:0) and PC(O-18:0/18:1), belonging to PC-O are selected by the xgboost model and are used to separate AS from HC (**Figure 2G**). From previous analysis we found serum levels of the PC-O were reduced in AS. As we known, PC was an important structural lipid in all cellular membranes, and PC-O was formed from the hydrolytic action of enzymes phospholipase A2, which was found in reasonable abundance in epithelial cells (19). Meanwhile, budding is an essential step in the life cycle of enveloped viruses, and enveloped viruses acquire their lipid membrane from the cells from which they bud (20). Therefore, deficiency of such PC lipids in AS may be the outcome of virus budding.

We discovered four classes of lipids with significant differences in SM and AS. Among them, the dysregulation of GP and SP could result in a lipotoxic insult relevant to the pathophysiology of common metabolic diseases (21). Li et al. discovered that the patients with metabolic diseases were at greater risk of developing into severe infections, and these comorbidities could also have an effect on the prognosis of COVID-19 (22). In addition, there existed an interaction between the platelet activating factor (PAF) and sphingolipid biosynthetic pathways, which derived from a CoA independent transacetylase, mediating the transfer of an acetyl group from PAF in the synthesis of N acetylsphingosine (23–25). The PAF is an ether-glycerosphospholipid that is important for immune cell activation and plays a critical role in chronic inflammation, virus infection, and immune activation (26). PE (18:1/16:0, 16:0/22:4 and 22:6/16:0) was one of the leading important lipids in SM and AS. PS showed statistical significance in the 28-subclass analysis. It was shown that PE synergized with PS to enhance PS receptor-mediated virus entry (27). The previous study suggested that the liposomes containing both PS and PE showed greater efficiency on the inhibition of the cells infected with a virus, such as Zika virus and Ebola, compared with the liposomes mixture separately composed of PS and PE (28). In addition, our study found that 9,10-dihydroxyoctadecenoic acid (9,10-DiHOME) decreased in symptomatic infections compared with asymptomatic infections (**Figure 3G**), and the importance of 9,10-DiHOME was also verified in machine learning. Lu et al. found that 9,10-DiHOME was altered in patients with HBV-related liver diseases and derived from the cytochrome P450 (CYP450) pathways (29).

Through analysis of the severe infections and moderate infections, 6-keto prostaglandin F1alpha (6 keto-PGF1α) was found to have the highest fold change among the compounds and the higher importance scores in machine learning. The deficiency of prostaglandin receptor may exacerbate the weight loss and delay in the viral clearance in the rats infected with the respiratory syncytial virus (30). Besides, the murine model infected by HSV discovered that 6-keto-PGF1α had a correlation with HSV-infection and cytotoxicity, and HSV-infection could suppress the release of 6-keto-PGF1α (31). Lysophosphatidylcholie (LPC) (12:0/0:0) had the highest VIP value and smallest fold change in SD and MD. Lysophosphatidylcholine had the function of reversibly arresting pore expansion during syncytium formation mediated by diverse viral fusogens (32). In addition, LPC and some saturated fatty acids could induce dendritic cell maturation, and the dendritic cells showed impaired functionality in COVID-19 (33, 34). Therefore, combined with the decreased expression level of LPC in SD compared with MD, it was suggested that the function and maturation of dendritic cells was destroyed in severe infections.

For LTNP vs STNP, we detected four classes including six subclasses with a total of 15 DELs. For example, GL subclasses TG upregulated in LTNP, whereas FA subclasses FFA downregulated. GL is a major form of energy storage (mainly in the form of TG) (35). As a hydrophobic component of the cell membrane, it plays an important role in structural function and has a dynamic impact on the signal mediators in intracellular and distant tissues (35, 36). TG is associated with cardiovascular disease, atherosclerotic cardiovascular disease, and ischemic heart disease (37–39). It has been reported that increased TG levels have also been observed in patients with inflammatory disease and HIV-infected patients (40, 41). Epidemiological studies have shown that elevated TG levels are independently associated with increased cardiovascular risk. Therefore, high intensity of TG might reflect pre-existing cardiovascular disease in LTNP which lead to the course time of COVID-19 more longer (42, 43). FA are carbon chains with methyl groups at one end and carboxyl groups at the other end, and they are also the first line of defense against pathogens. Fatty acids are essential for viral infection because they provide the basis for various membrane lipids during viral proliferation, which plays an important role in immune and inflammatory responses (44, 45). In our data, FFA downregulated in LTNP (**Figure 5F**), and thus dysfunctional immune or inflammatory responses might be the cause of positive long-term nucleic acid tests. In conclusion, our results suggest that the prolonged nucleic acid conversion time may be due to the patient’s own underlying disease or immune dysregulation.

Furthermore, this study has some limitations. Firstly, there is a lack of follow-up on AS. Secondly, the study lacks subsequent experiments on cytokines. We plan to further conduct basic research based on our significant findings. Lastly, there is a lack of longitudinal studies on infected individuals. We hope that future research can delve deeper into these areas.

In conclusion, our study presents a systematic lipidomic investigation of serum samples from 220 plasma samples of 62 healthy controls and 104 COVID-19 patients and found multiple lipids that were associated with the virus infection, disease severity, and the viral persistence of the COVID-19 patients. Our findings may help us understand the potential contributions of lipid metabolites to the pathogenesis of COVID-19. We have demonstrated the potential of identifying novel biomarkers for the diagnosis and treatment of SARS-CoV-2 infection based on analysis of five compared groups of lipid metabolites. In conclusion, our results may provide useful diagnostic and therapeutic clues for COVID-19.

## Data availability statement

The datasets presented in this study can be found in online repositories. The names of the repository/repositories and accession number(s) can be found below: MTBLS7937 (Metabolights).

## Ethics statement

The studies involving human participants were reviewed and approved by the First Affiliated Hospital of Xi’an Jiaotong University (XJTU1AF2020LSK-015) and the Renmin Hospital of Wuhan University (WDRY2020-K130). The patients/participants provided their written informed consent to participate in this study.

## Author contributions

Conceptualization: CZ and BS. Methodology and Investigation: XL, XQ, JY, XW, HQ, FH, HB and YL. Resources: CZ, BS, BHZ, and KH. Writing: XL and XQ. Review and editing: XL, XQ and BS. Supervision: BS and JY. Funding: BS. Project administration: BS. All authors contributed to the article and approved the submitted version.

## Supplementary material

Supplementary Figure 1
**(A)** The raw intensity of FA, GL, GP, SP and ST classes in 5 compared groups. **(B)** The raw intensity of 28 subclasses for lipids in LTNP vs STNP. **(C)** The heatmap of fold change for FA, GL, GP, SP and ST classes in 5 compared groups; **(D)** the rising sun map of 732 lipids in 28 subclasses and 5 classes.

Supplementary Figure 2 The raw intensity of different degrees of unsaturation for FA, GL, GP, SP and ST classes in 3 compared groups, including AS vs HC **(A)**, SM vs AS **(B)** and LTNP vs STNP **(C)**.

Supplementary Figure 3 The raw intensity of different carbon chain lengths of FA, GL, GP, SP and ST classes in 5 compared groups. The lipids of each class are defined according to our data as follows, FA: short chains (C<12), medium chain (C=12 or C=14), long chain (C>14); GL: short chain (C<32), medium chain (C=32 or C=34), long chain (C>34); GP: short chain (C<32), medium chain (C=32 or C=34), long chain (C>34); GL: short chain (C<32), medium chain (C=32 or C=34), long chain (C>34); SP: short chain (C<32), medium chain (C=32 or C=34), long chain (C>34); ST: short chain (C<18), medium chain (C=18 or C=20), long chain (C>20).

Supplementary Figure 4 Receiver operating characteristic (ROC) and performance of the xgboost model in the test set for 5 compared groups.

Supplementary Figure 5 The KEGG analysis of the differentially expressed lipids in 5 compared groups.

Supplementary Table 1 the detailed characteristic information of samples used in this study.

Supplementary Table 2 Summary of lipids in this study.

Supplementary Table 3 the differentially expressed lipids in 5 compared groups.

Supplementary Table 4 the KEGG results for differentially expressed lipids in 5 compared groups.
