# Supplementary figures and images for: Lipid alternations in the plasma of COVID-19 patients with various clinical presentations
Source: Front Immunol. 2023 Aug 29;14:1221493. doi: 10.3389/fimmu.2023.1221493 (PMC10495680; doi:10.3389/fimmu.2023.1221493)

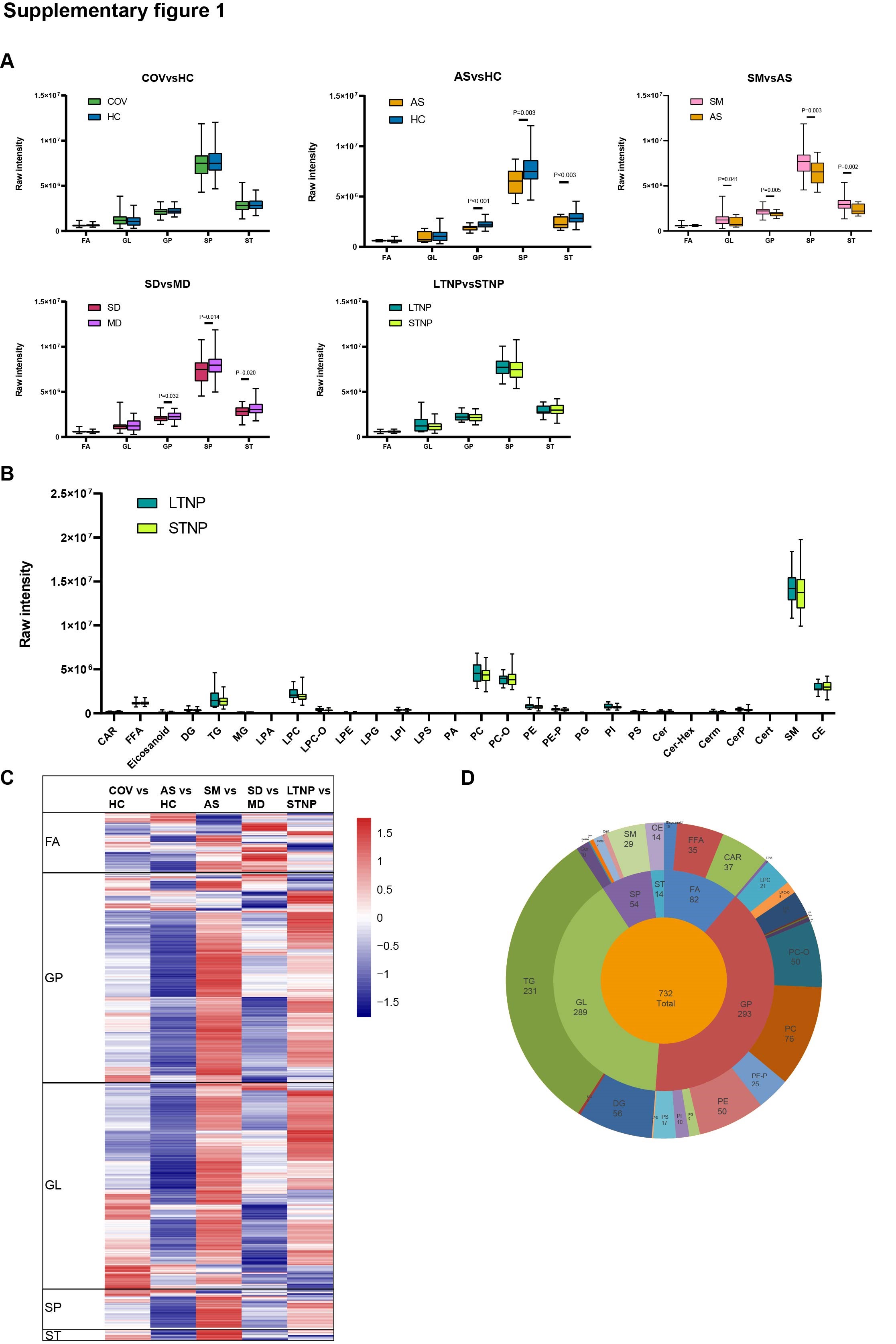

Supplement: Supplementary Figure 1 — (A) The raw intensity of FA, GL, GP, SP and ST classes in 5 compared groups. (B) The raw intensity of 28 subclasses for lipids in LTNP vs STNP. (C) The heatmap of fold change for FA, GL, GP, SP and ST classes in 5 compared groups; (D) the rising sun map of 732 lipids in 28 subclasses and 5 classes. [file Image_1.jpg]

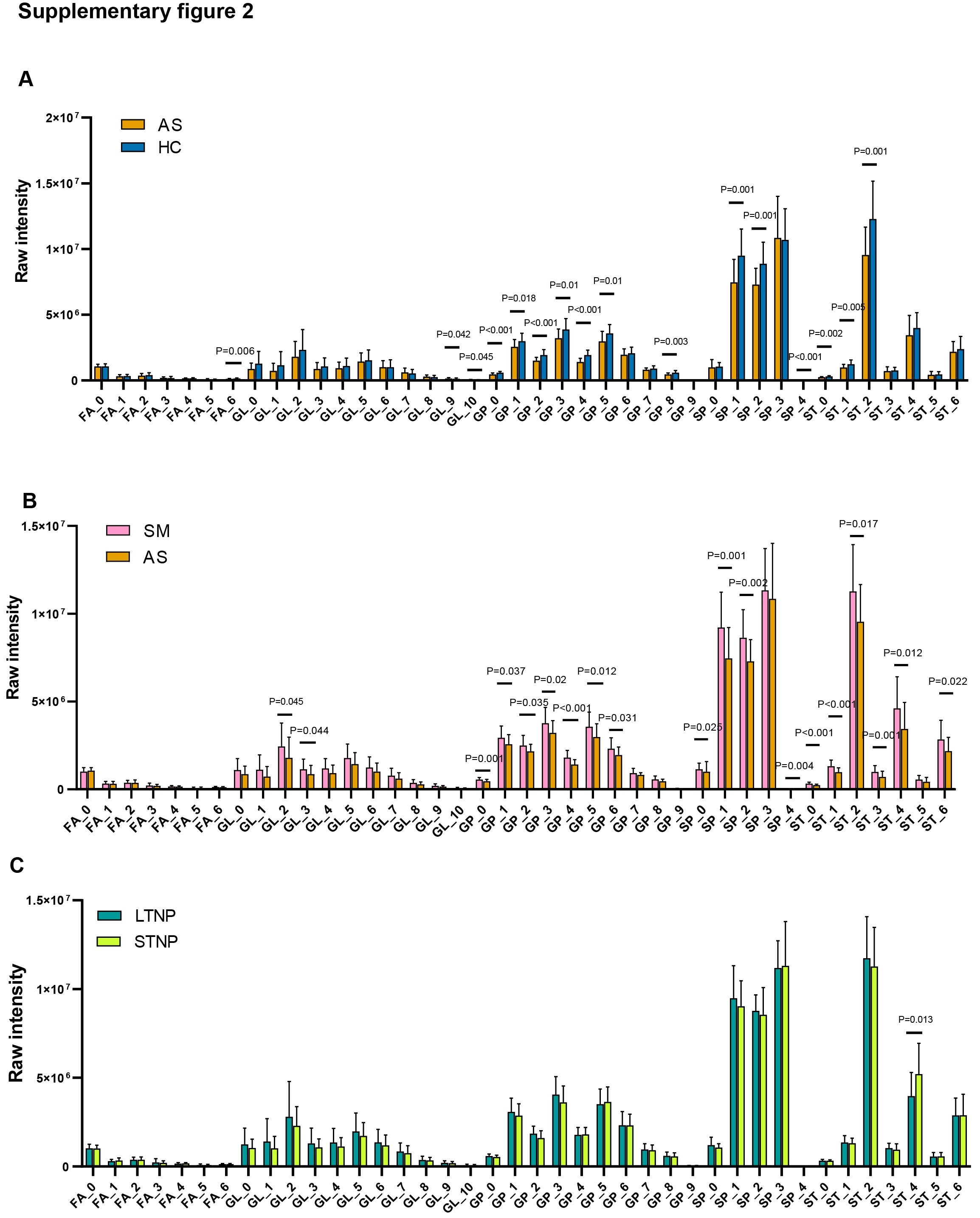

Supplement: Supplementary Figure 2 — The raw intensity of different degrees of unsaturation for FA, GL, GP, SP and ST classes in 3 compared groups, including AS vs HC (A), SM vs AS (B) and LTNP vs STNP (C). [file Image_2.jpg]

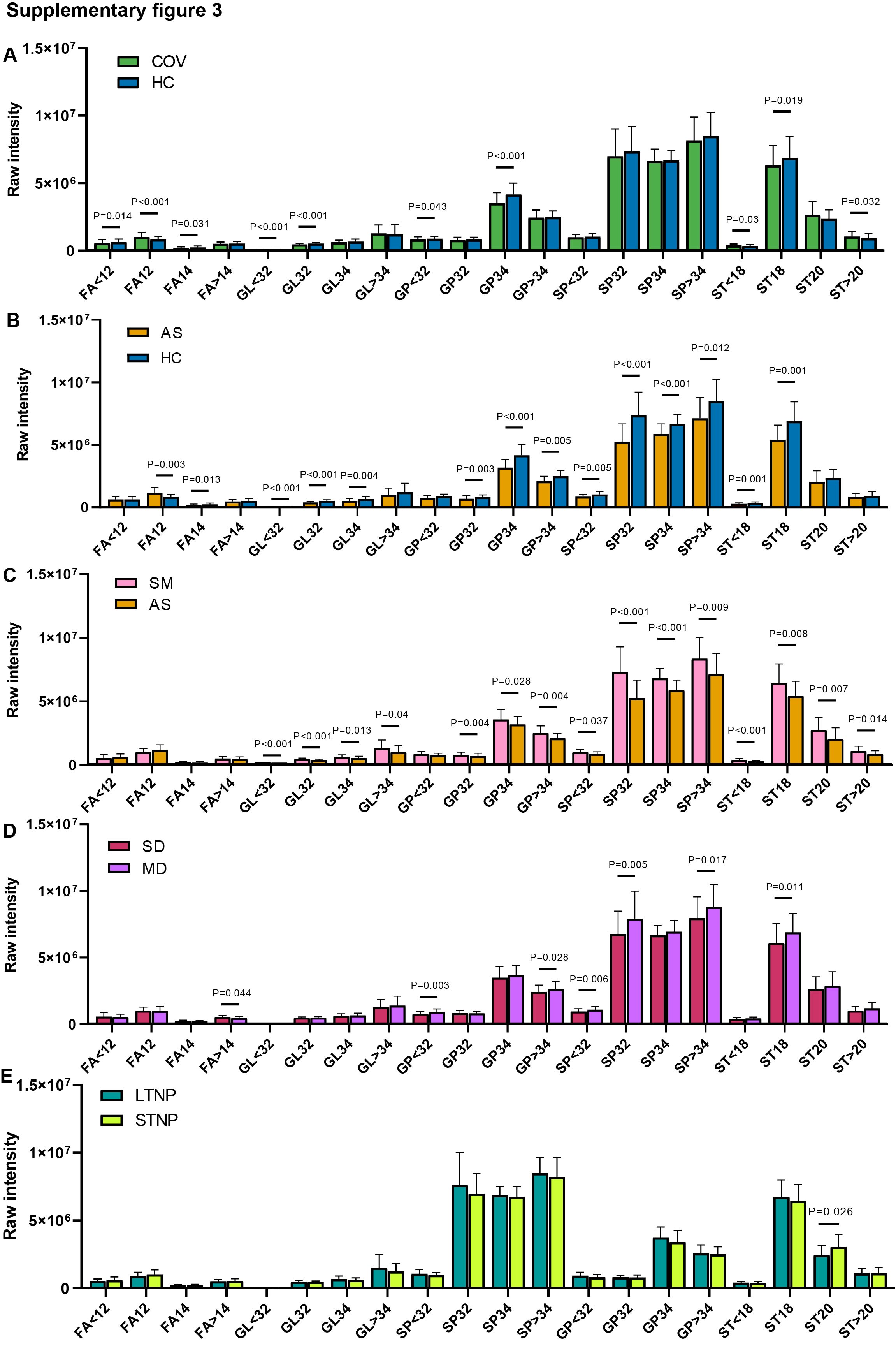

Supplement: Supplementary Figure 3 — The raw intensity of different carbon chain lengths of FA, GL, GP, SP and ST classes in 5 compared groups. The lipids of each class are defined according to our data as follows, FA: short chains (C<12), medium chain (C=12 or C=14), long chain (C>14); GL: short chain (C<32), medium chain (C=32 or C=34), long chain (C>34); GP: short chain (C<32), medium chain (C=32 or C=34), long chain (C>34); GL: short chain (C<32), medium chain (C=32 or C=34), long chain (C>34); SP: short chain (C<32), medium chain (C=32 or C=34), long chain (C>34); ST: short chain (C<18), medium chain (C=18 or C=20), long chain (C>20). [file Image_3.jpg]

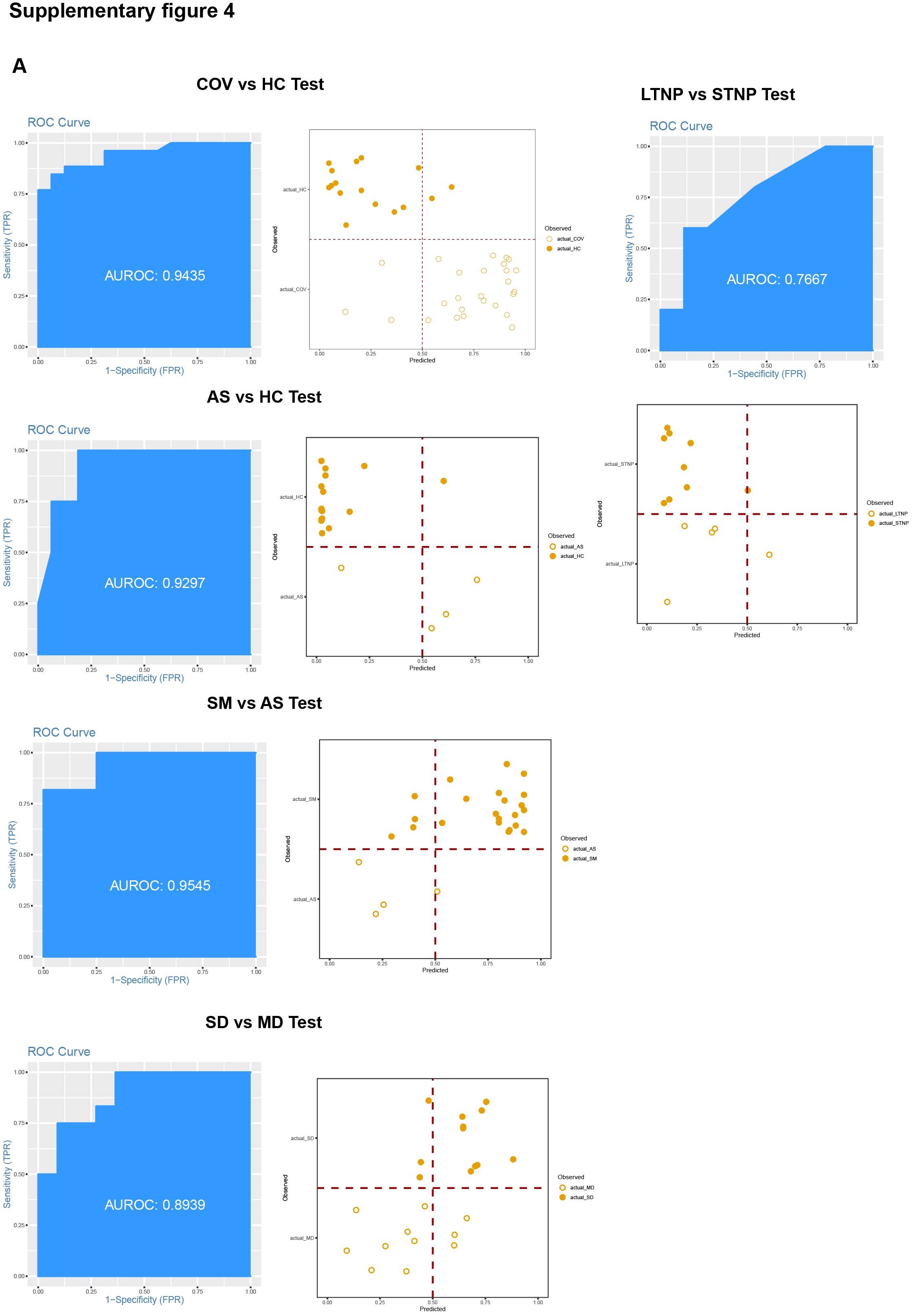

Supplement: Supplementary Figure 4 — Receiver operating characteristic (ROC) and performance of the xgboost model in the test set for 5 compared groups. [file Image_4.jpg]

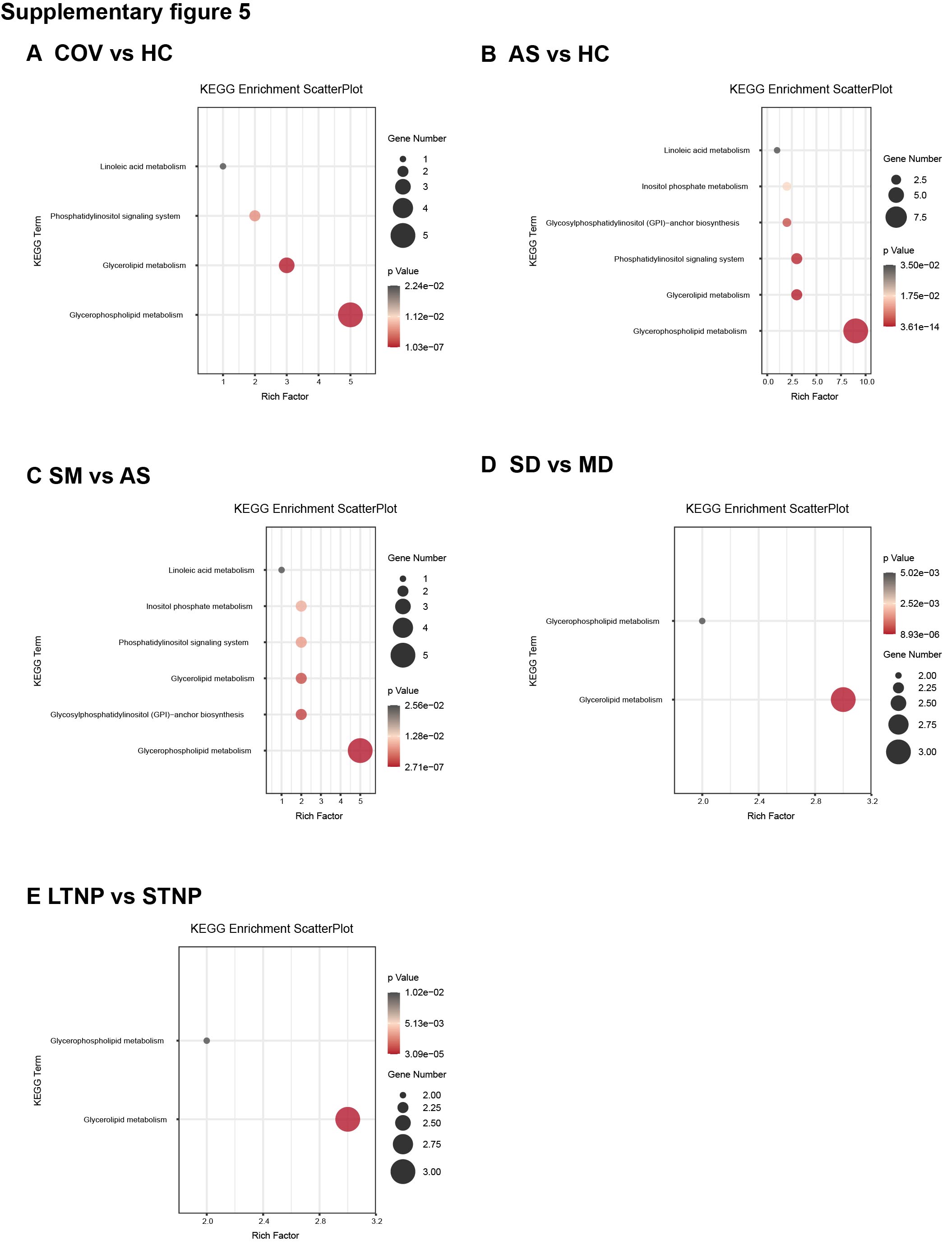

Supplement: Supplementary Figure 5 — The KEGG analysis of the differentially expressed lipids in 5 compared groups. [file Image_5.jpg]
